# Supplementary material for: Nutritional redundancy in the human diet and its application in phenotype association studies
Source: Nat Commun. 2023 Jul 18;14:4316. doi: 10.1038/s41467-023-39836-0 (PMC10354046; doi:10.1038/s41467-023-39836-0)
Supplement: Supplementary file 1 — Supplementary Information [file 41467_2023_39836_MOESM1_ESM.pdf]

Nutritional Redundancy in the Human Diet and its Application in Phenotype association studies

Supplemental Information

1. NR calculating using human dietary data **2**
1.1 Reference FNN 2
1.2 Nutrition profile analysis 3
1.3 Food Choice Analysis 3

2. Healthy diet scores **3**
2.1 HEI-2005 3
2.2 AHEI-2010 3
2.3 AMED 3
2.4 DASH 4

3. Healthy aging prediction **4**

4. Supplementary figures and tables **5**

1. NR calculating using human dietary data

1.1 Reference FNN

1.1.1 USDA database

To construct the Food-Nutrient network, we downloaded the FNDDS 2011-2012 from the USDA database, which including 7,618 foods and 65 macronutrients. The USDA National Nutrient Database for Dietary Studies is the major source of food composition data in the United States. To be consistent with the version used in DMAS study, we chose the version 2011-2012 to construct reference Food-Nutrient network (FNN). This version includes 7,618 foods, which can be clarified into 9 highest items and the total number of nutrients is 65.

Remark 1: In calculating the nutritional profiles (Fig.1) as well as the nutritional redundancy (Fig.4), we excluded energy and water for the following considerations. First, energy does not have a unit of mass, and hence cannot be included in the nutritional profile where components represent relative abundances. Consequently, it cannot be used to calculate the nutritional redundancy either. Second, water was not considered as a nutrient in the Harvard Food Composition Database (HFDB). For consistency and comparison purposes, we also removed it from FNDDS when we calculate the nutritional profiles and nutritional redundancy from the DMAS, WLVS, MLVS data.

1.1.2 Frida database

The database Frida Food Data (frida.fooddata.dk) was created and published by the National Food Institute, Technical University of Denmark (DTU), including data on nutrient content of various foods. We used the version released at 08-02-2019, which includes 1,185 foods items and 198 nutrients.

1.1.3 Harvard Food Composition Database

We used the Harvard food composition table of year 2015 to construct a reference food-nutrient network to calculate the nutritional redundancy of participants from the NHS. The Harvard food composition table consists of 575 foods and 182 nutrients. To calculate the nutritional redundancy, we removed calories due to different unit and total protein, total fat and total sugar since those nutrients are overlapped with some sub-nutrients.

1.1.4 Other databases

Other databases, for example, FooDB, a database representing the most comprehensive effort to integrate food composition data from specialized databases and experimental data, has provided the information of 26,625 distinct bio-chemicals in foods. PhenolExplorer and eBasis have also resulted in wealth of information on food composition. Throughout our analysis, we focused on the nutrient level, rather than composition and compounds levels, thus we reference FNN are constructed by USDA and Frida databases.

1.2 Nutrition profile analysis

The nutritional composition of foods for DMAS was determined using ASA24-2016. Both of WLVS and MLVS composition of foods for DMAS was determined using ASA24-2012. ASA24 assigns nutrient information to foods using the USDA's Food and Nutrient Dietary Database (FNDDS). Subjects reported dietary intake as food records and entered their own dietary records directly into ASA24. To calculate the relative abundance of nutrient, we removed energy (unit is not in mass) and water (the water information is not included in NHS database, so we removed to be consistent).

1.3 Food Choice Analysis

Foods of DMAS, WLVS and MLVS were categorized according to their FNDDS food code and modification code as assigned by ASA24. The foods of NHS were mapped from serving data to the Harvard food composition table according to the food descriptions.

2. Healthy diet scores

2.1 HEI-2005

HEI-2005[1] is a score that measures adherence to the USDA 2005 Dietary Guidelines for Americans. The score range is 0 to 100. Each of the 12 components has a minimum score of zero and a maximum score of 5, 10 or 20. These components are: Total vegetables, Dark green & orange vegetables, Total fruit, Whole fruit, Total grains, Whole grains, Milk, Oils, Saturated fat, Sodium and SoFAAs.

2.2 AHEI-2010

AHEI-2010[1] is a score that measures adherence to a diet pattern based on foods and nutrients most predictive of disease risk in the literature. The minimum score = 0, maximum score = 110. Each of the 11 components has a minimum score of 0 and a maximum score of 10, as outlined in the table below. A score between the minimum and maximum is assigned on a continuous basis (except for sodium and alcohol). Those 11 components are: Vegetables, Fruit, Whole grains, Sugar-sweetened beverages and fruit juice, Nuts and legumes, Red meat and processed meat, Trans fat, Long-chain fats, Poly-unsaturated fatty acids, Sodium and Alcohol.

2.3 AMED

The components of AMED[2] are vegetables (excluding potatoes), fruits, nuts, whole grains, legumes, fish, ratio of monounsaturated to saturated fat, red and processed meats, and alcohol. The range of score is 0 to 9. The score criteria are: Intake above the FFQ-specific median intake received 1 point for vegetables, fruits, nuts, whole grains, legumes, fish, and M:S ratio; otherwise, they received 0 points. Red and processed meat consumption below the FFQ-specific median

received 1 point; otherwise, 0 points. Alcohol intake between 5 and 15 g/d for women and 10-25 g/d for men received 1 point; otherwise, 0 point.

2.4 DASH

This score was created to capture the characteristics of the Dietary Approaches to Stop Hypertension diet. The DASH[3] Components includes fruits, vegetables (excluding potatoes), nuts and legumes, low-fat dairy products, whole grains, sodium, sweetened beverages, red and processed meats. The score range is 8 to 40. The DASH Scoring Criteria is each food group first classified into FFQ-specific quintiles. For fruits, vegetables, nuts and legumes, low-fat dairy products, and whole grains, the score for that food group is the quintile ranking. i.e., quintile 1 is assigned 1 point and quintile 5, 5 points. For sodium, red and processed meats, and sweetened beverages, low intake is best. Therefore, the lowest quintile was given a score of 5 points and the highest quintile, 1 point.

3. Healthy aging prediction

We used two standard classifiers: RF (Random Forest, R package 'randomForest'[4]) and XGBoost (Extreme gradient boosting decision trees, R package 'xgboost'[5]) to predict the healthy aging status. The base learners of RF are decision trees. Each tree is a non-linear model constructed with many linear boundaries. A node in a decision tree is associated with a question asking about the data based on the value of a particular feature. XGBoost is a scalable end-to-end decision tree boosting system[5]. Unlike RF that applies the technique of bootstrap aggregating (i.e., bagging) to tree learners, the trees of a boosting system are built sequentially: each tree aims to reduce the error of its previous tree.

For hyperparameter tuning, we used the R package 'caret'[6] (Classification And REgression Training). The number of features randomly sampled as candidates at each split range from 1 to 15 and the number of trees to grow is fixed to 500 for RF. The parameter ranges for XGBoost are the following: (1) Learning rate (eta): 0.001, 0.01; (2) Number of features supplied to a tree (colsample_bytree): 0.4, 0.6, 0.8, 1.0; (3) The depth of the tree (max_depth): 4, 6, 8, 10, 100,000; (4) Maximum number of iterations (nrounds): 100, 1000; (5) Regularization (gamma): 0; (6) Minimum sum of instance weight (min_child_weight): 1; (7) Number of samples supplied to a tree (subsample): 0.5, 0.75, 1; (8) Number of trees to grow (ntree): 500.

To overcome the label imbalance issue, we used the downSample function in caret, which will randomly sample a data set so that all classes have the same frequency as the minority class. To compare the performances of NR and the four healthy diet scores, we split the data into 80% percentage of sample as training set and the remaining 20% samples as test set. For each splitting, we used one of NR, HEI-2005, AHEI-2010, AMED and DASH together with other confounding factors to train the model, then validate the classifier using the test set. We used the error rate, i.e., the proportion of participants that have been incorrectly classified by the model and AUC (area under the ROC curve) to quantify the performance.

4. Supplementary figures and tables

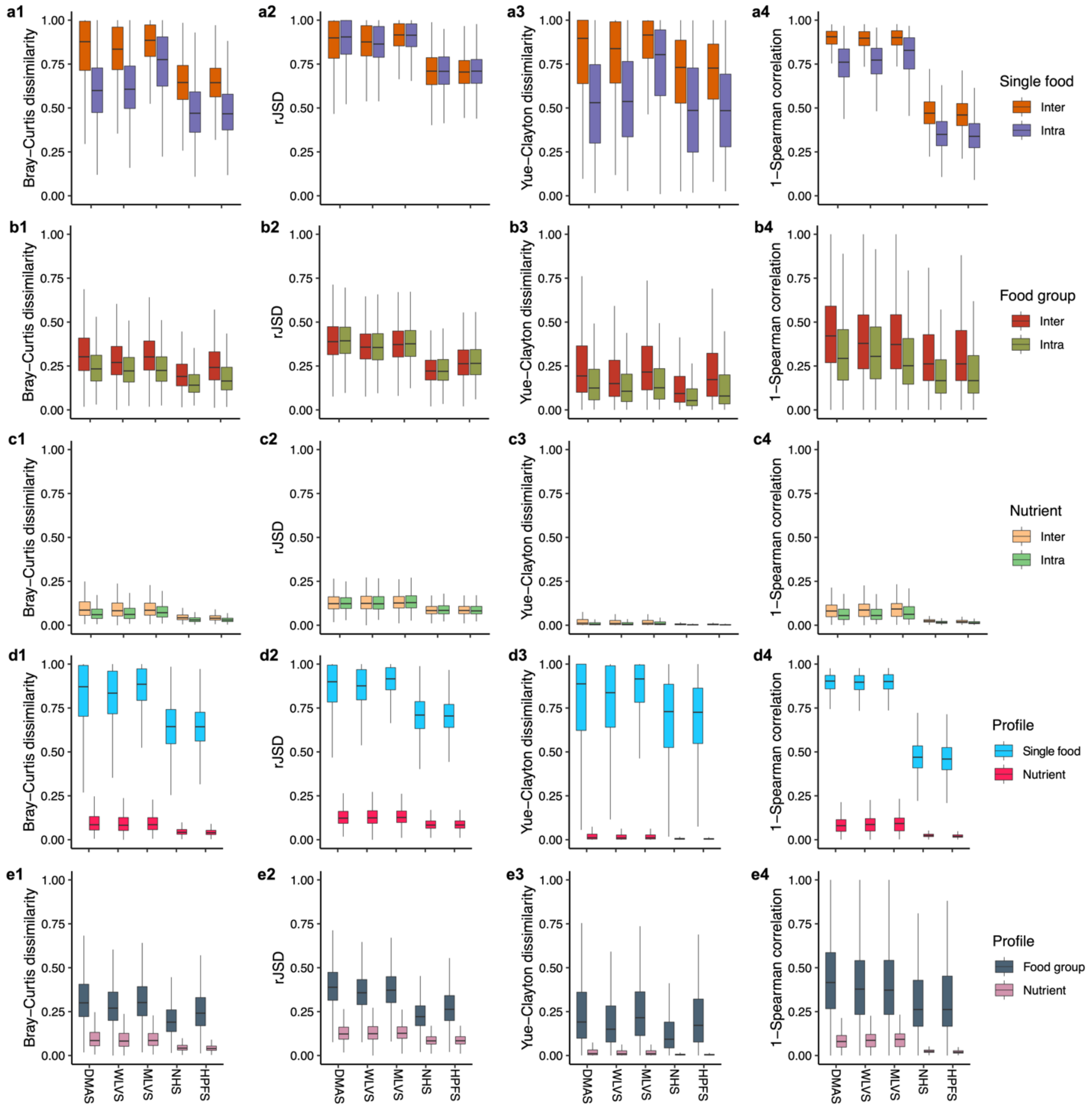

Figure S1: Nutritional profiles are highly conserved across individuals while food profiles are highly personalized. The Bray-Curtis dissimilarity (column-1), rJSD (rooted Jensen-Shannon divergence, column-2), Yue-Clayton distance (column-3) and 1-Spearman correlation (column-4) between the food profiles of the same individuals but different time points (intra-individual) and food profiles among different individuals (inter-individual) at single food level (a1-a4) and nine major food groups level (b1-b4) and nutrient profiles (c1-c4). The Bray-Curtis dissimilarity (column-1), rJSD (column-2), Yue-Clayton distance (column-3) and 1-Spearman correlation (column-4) between the food (or nutritional) profiles of different individuals and different time

points at the single food level (d1-d4) and nine major food group level (e1-e4). The Bray-Curtis dissimilarity between a pair of individuals, j and k is defined as: $BC_{jk} \equiv \frac{\sum_i |X_{ij}-X_{ik}|}{\sum_i (X_{ij}+X_{ik})}$. The rJSD dissimilarity is defined as: $D_{\mathrm{rJSD}}(j,k) \equiv [\frac{D_{\mathrm{KL}}(j,m)+D_{\mathrm{KL}}(k,m)}{2}]^{1/2}$, in which $m \equiv \frac{j+m}{2}$ and $D_{\mathrm{KL}}(j,k) \equiv \sum_{i\in S} X_{ij} \log \frac{X_{ij}}{X_{ik}}$ is the Kullback-Leibler divergence between j and k. The Yue-Clayton dissimilarity is defined as: $YC_{jk} \equiv \frac{\sum_i X_{ij}X_{ik}}{\sum_i \sum_i (X_{ij}-X_{ik})^2 + \sum_i (X_{ij} * X_{ik})}$. In all dissimilarity definitions, X_{ij} represents the relative abundance of food/nutrient i in individual j. We only choose 100 participants in NHS and HPFS due to computational complexity. The boxplot represents all pairwise dissimilarity. Boxes indicate the interquartile range between the first and third quartiles with the central mark inside each box indicating the median. Whiskers extend to the lowest and highest values within 1.5 times the interquartile range.

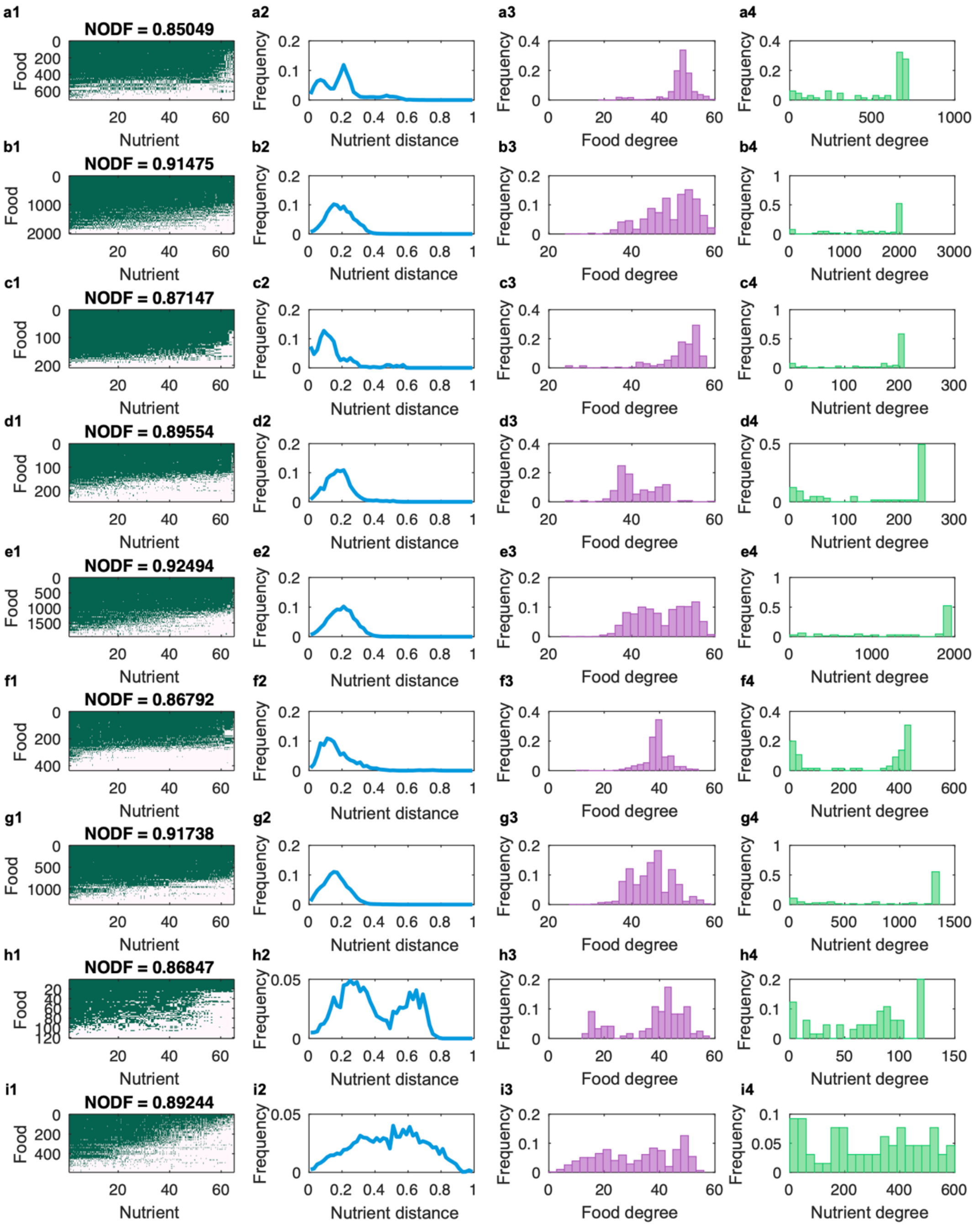

Figure S2: The food-specific subgraphs of the FNN display similar topological feature as the original FNN. Columns: (1) the incident matrix; **(2)** distribution of nutrient distance; **(3)** distribution of food degree; **(4)** distribution of nutrient degree. **Rows:** (**a**) Milk and Milk Products; (**b**) Meat_Poultry, Fish, and Mixtures; (**c**) Eggs; (**d**) Dry Beans, Peas, Other Legumes, Nuts, and Seeds; (**e**) Grain Products; (**f**) fruits; (**g**) Vegetables; (**h**) Fats, Oils, and Salad Dressings; (**i**) Sugars, Sweets, and Beverages.

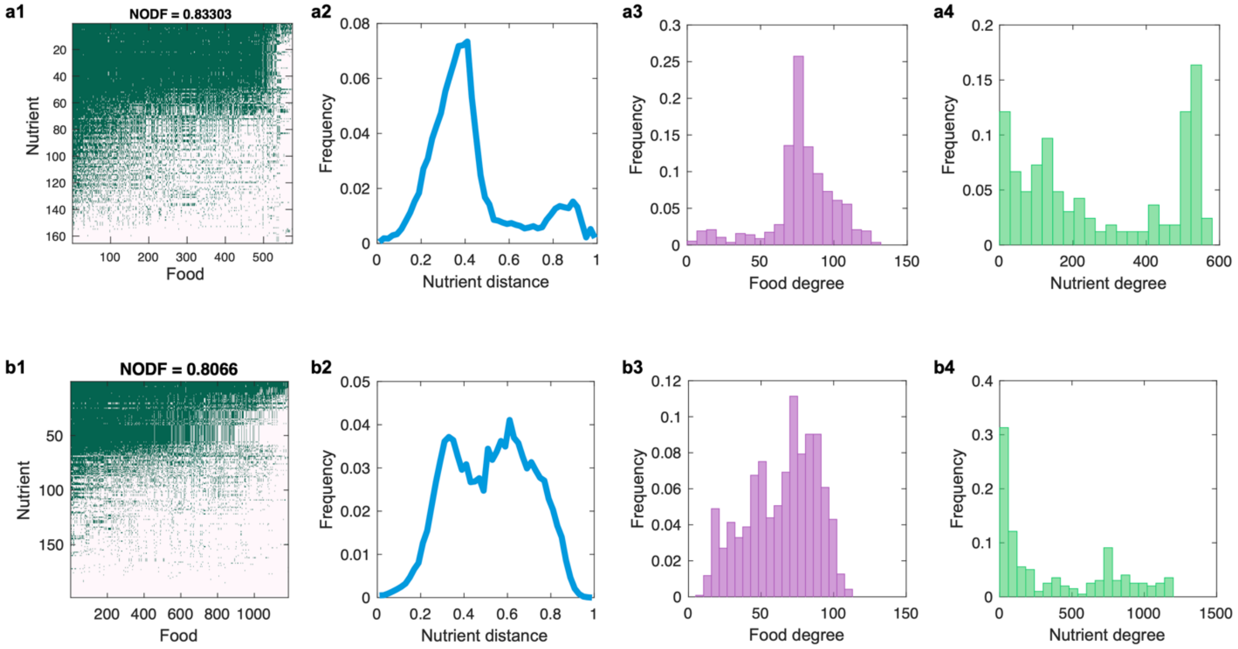

Figure S3: Food-Nutrient networks (FNN) constructed from the Harvard food composition database (a) and the Frida database (b). Columns: (**1**) the incident matrix of FNN, where the presence (or absence) of a link between a food and a nutrient is colored in green (or pink), respectively. (**2**) The distribution of nutrient distances (d_{ij}) among different foods. The bin size is 0. (**3**) The distribution of food degrees. Here, the degree of a food item is the number of distinct nutrients it can provide. (**4**) The distribution of nutrient degrees. Here, the degree of a nutrient is the number of food items that contain this nutrient.

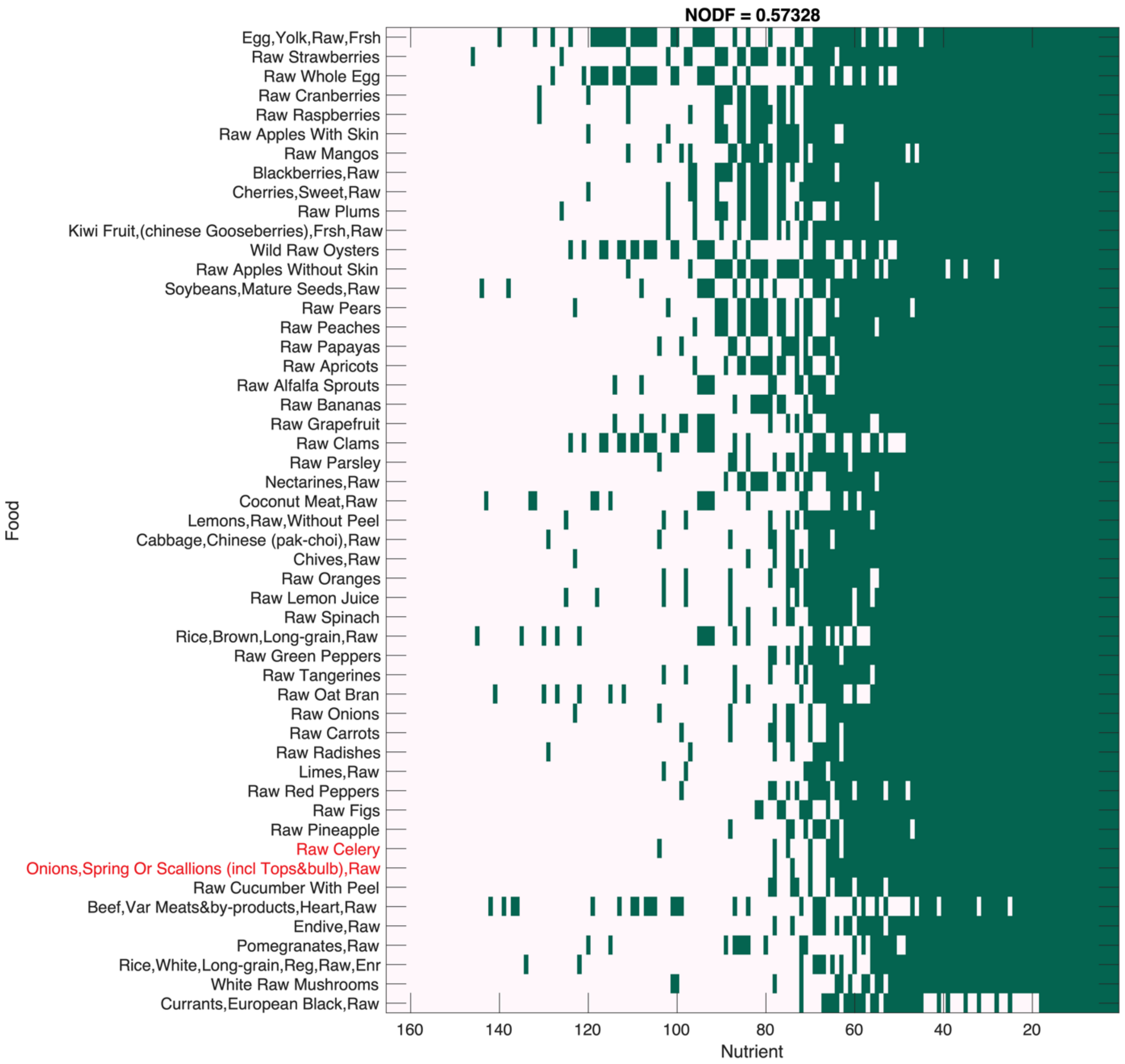

Figure S4: Raw food-Nutrient networks (FNN) constructed from the Harvard food composition database. We organized this matrix using the Nestedness Temperature Calculator to emphasize its nested structure[6].

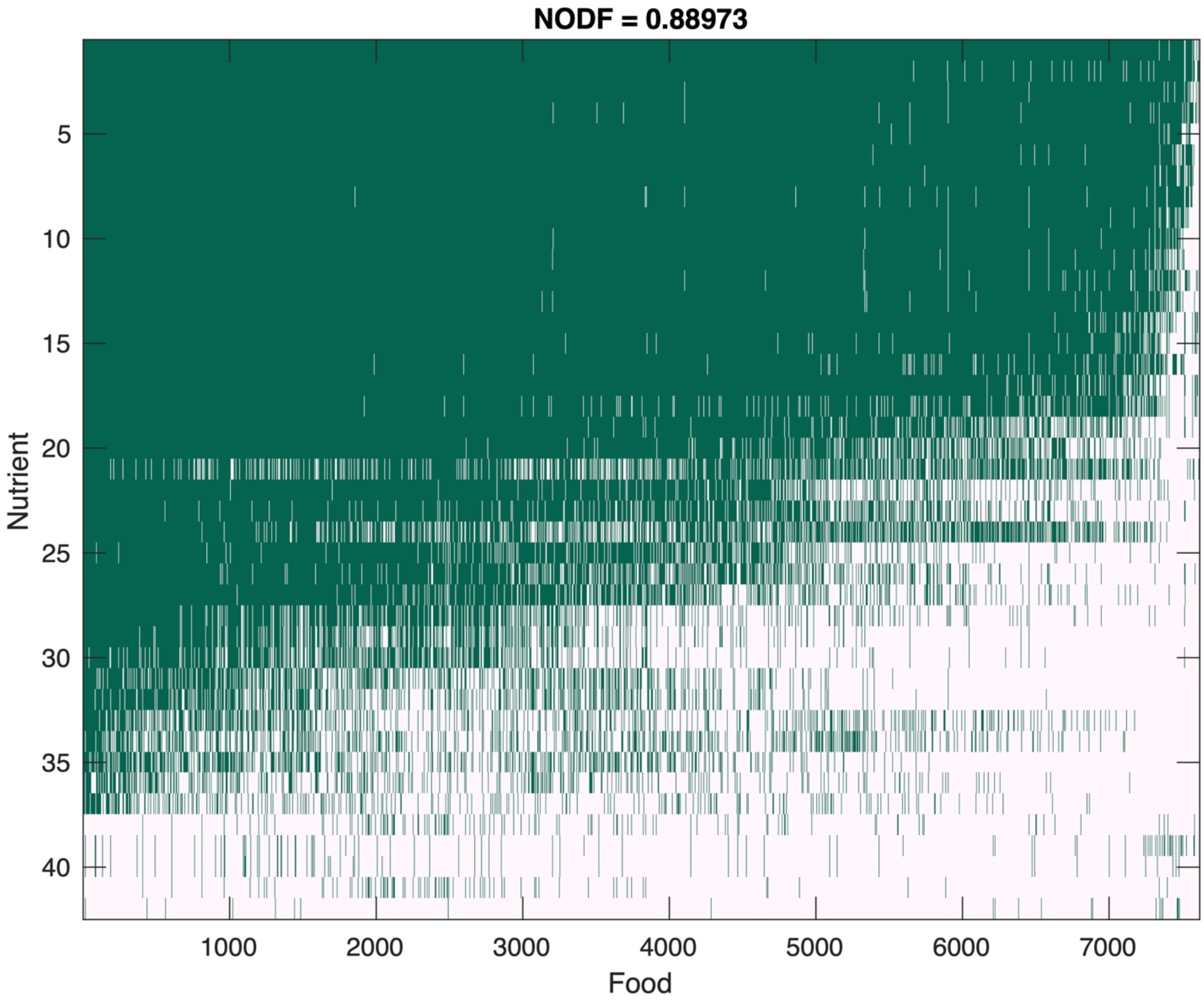

Figure S5: Food-Nutrient networks (FNN) constructed from the FNDDS database after removing those nutrients that are not specific enough to have a SIMLES or InChIKey ID, e.g., sugar, total fat, protein, total fiber, etc. We organized this matrix using the Nestedness Temperature Calculator to emphasize its highly nested structure[6].

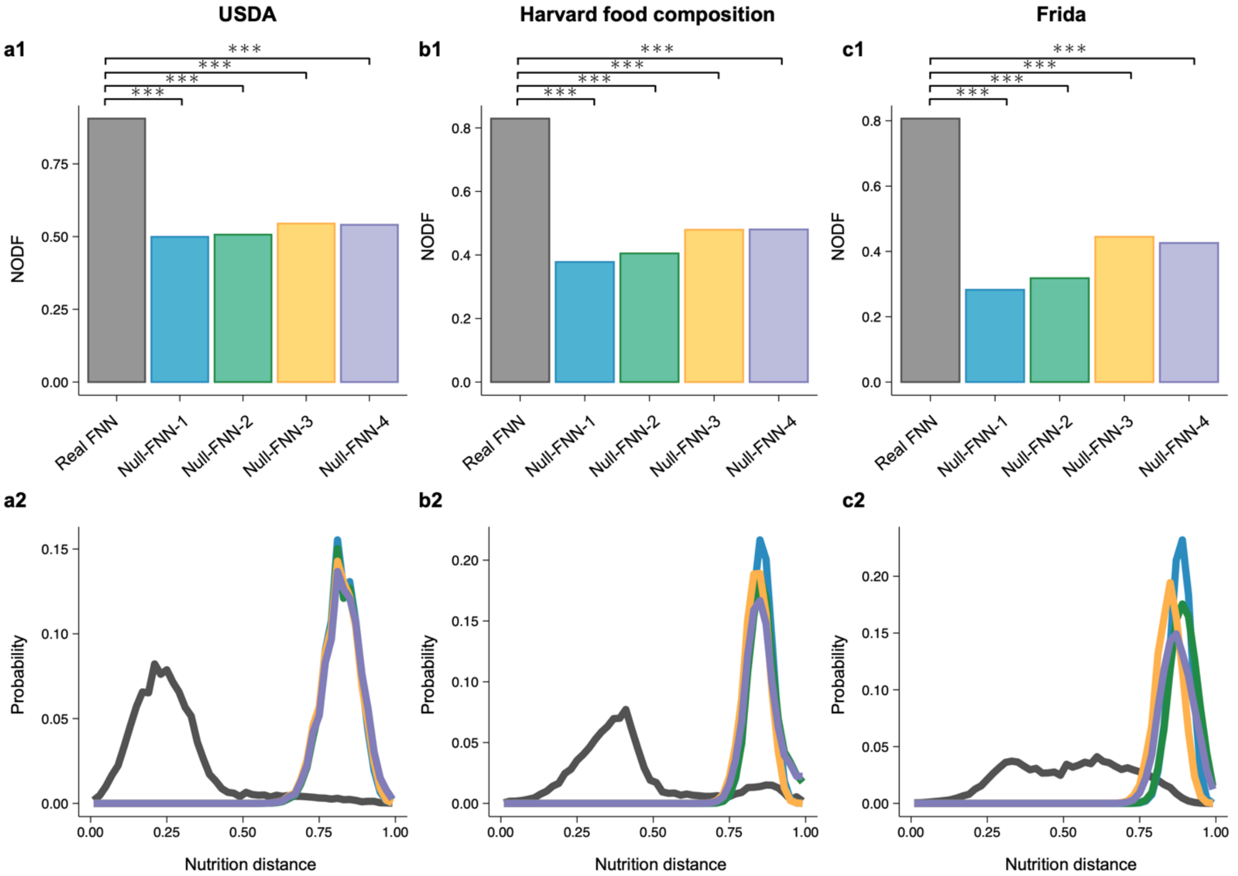

Figure S6: Comparison of structural properties between the real FNN and different randomized FNNs. **Columns**: FNN constructed from the USDA database **(a)**, the Harvard food composition database **(b)**, and the Frida database **(c). Rows: (1)** The nestedness based on the NODF measure of the real FNN (gray bar), as well as the randomized FNNs (colored bars) using four different FNN randomization schemes: Null-FNN-1, complete randomization; Null-FNN-2, Food-degree preserving randomization; Null-FNN-3, Nutrient-degree preserving randomization; Null-FNN-4, Food- and nutrient-degree preserving randomization. For each randomization scheme, 50 realizations were generated. **(2)** The distribution of nutrient distances (d_{ij}) between different foods calculated from the real FNN (gray lines) and the randomized FNNs (colored lines) using the same randomization schemes as in row (1). We generated 50 realizations for each randomization scheme, and the bin size is 0.02. All FDR-corrected P values were found using the paired and two-sided t-test. Significance level: FDR-corrected p value <0.0001(***).

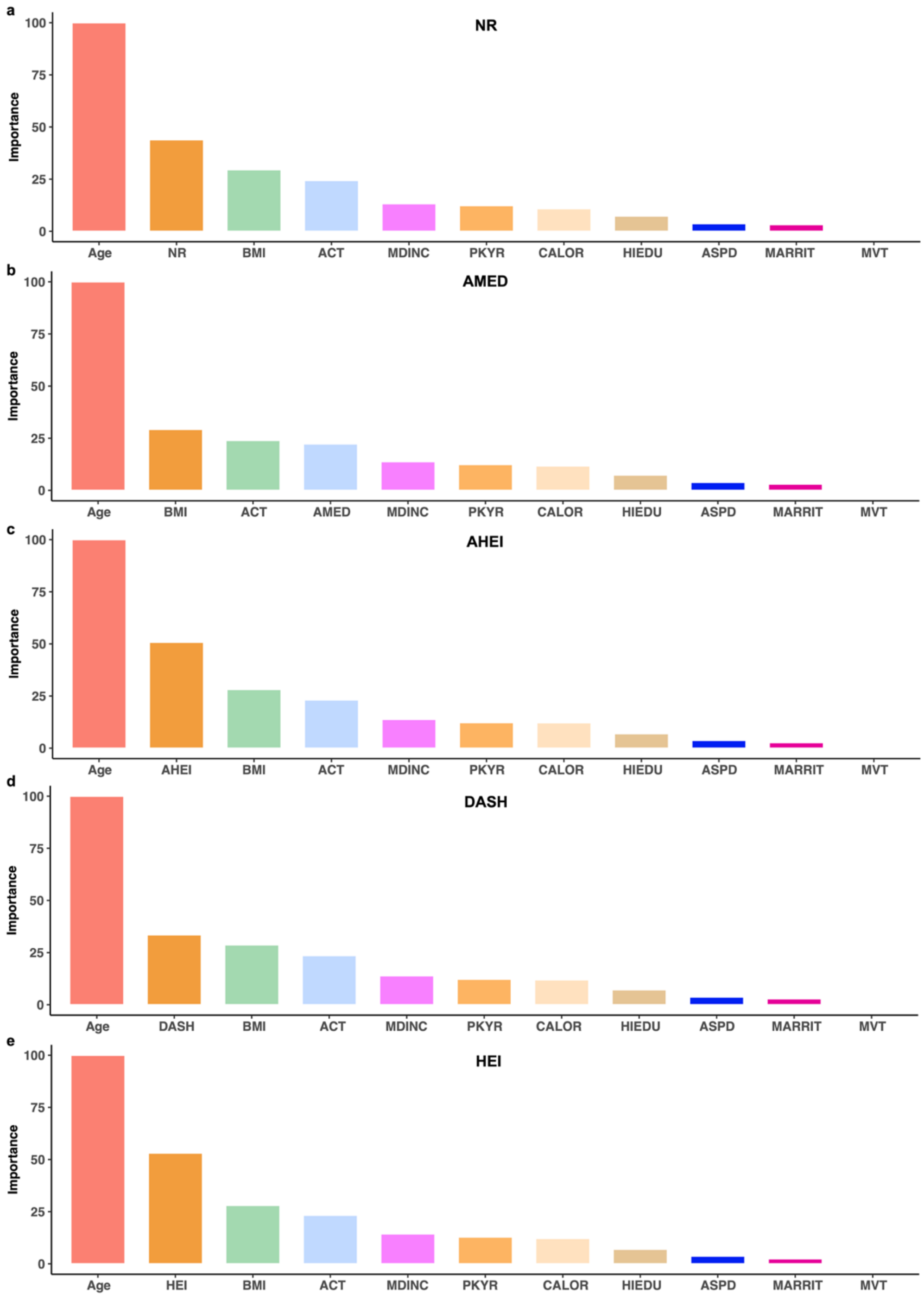

Figure S7: Importance ranking of features quantified by their mean decreasing of accuracy. Here, we used the following features: Age, BMI (body mass index), ACT (physical activity), MDINC (median income from census tract), CALOR (energy intake), PKYR (pack-years of smoking), HIEDU (education), ASPD (aspirin use), MVT (multivitamin use), MARRIT (marital status), together with (**a**) NR (nutritional redundancy) or one of the healthy diet scores: (**b**) AMED; (**c**) AHEI; (**d**) DASH; and (**e**) HEI. The results were averaged over 100 different training/test splits. The error bars are almost invisible in those figures.

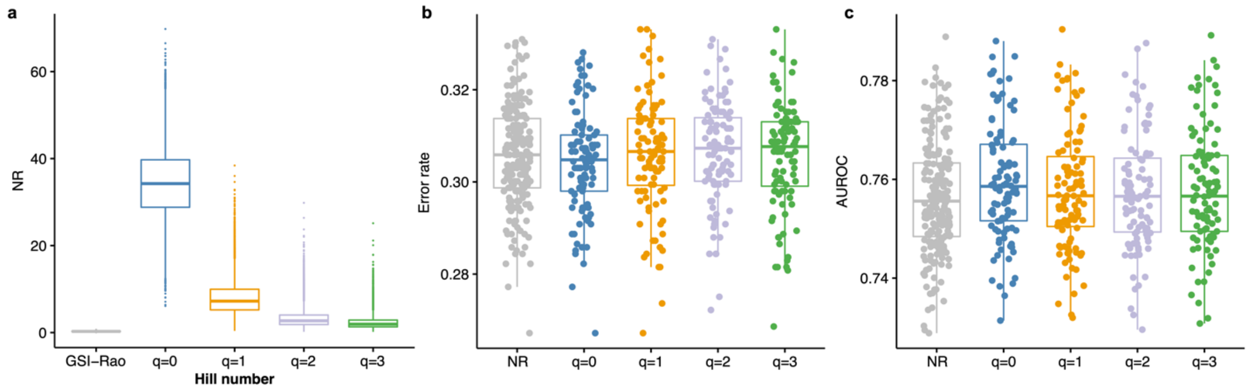

Fig. S8: Nutritional redundancy is robust in aging prediction with different Hill numbers. a: NR of NHS calculated with different definitions. GSI-Rao represents the NR calculated from Gini-Simpson index and Rao's quadratic entropy used in the main text. $q = 0,1,2,3$ represent the Hill-number based NR (see SI Sec.3.3). **b:** Error rate of the RF classifier in predicting the healthy aging status. **c:** AUROC of the RF classifier in predicting the healthy aging status. The participants are randomly spitted into 80% as the training set and the remaining 20% as the test set. The boxplot represents the performances of 200 independent splitting. Boxes indicate the interquartile range between the first and third quartiles with the central mark inside each box indicating the median. Whiskers extend to the lowest and highest values within 1.5 times the interquartile range.

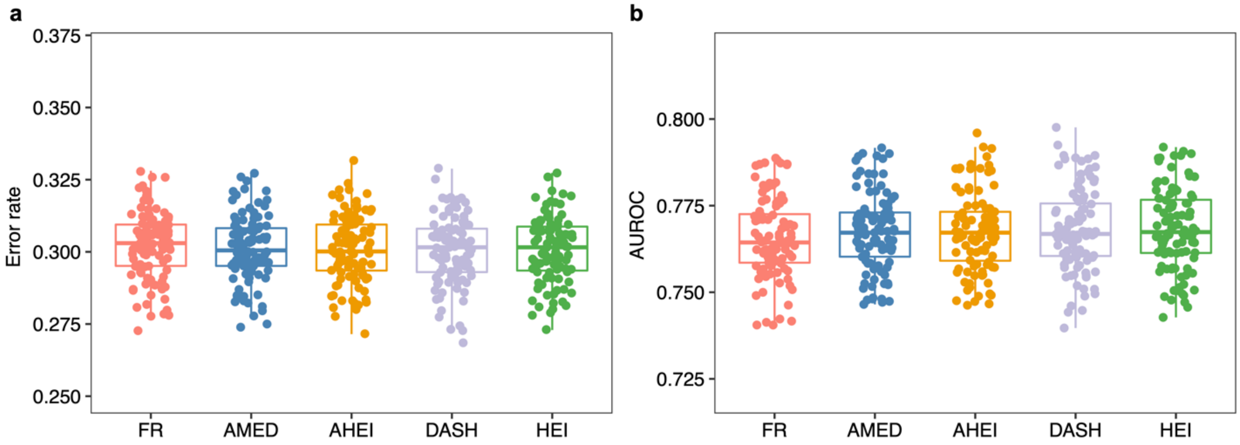

Fig. S9: Nutritional redundancy is robust in predicting healthy aging with a different classifier. a: Error rate of the XGBoost classifier in predicting the healthy aging status. **b:** AUROC of XGBoost in predicting the healthy aging status. The participants are randomly spitted into 80% as the training set and the remaining 20% as the test set. The boxplot represents the performances of 100 independent splitting. Boxes indicate the interquartile range between the first and third quartiles with the central mark inside each box indicating the median. Whiskers extend to the lowest and highest values within 1.5 times the interquartile range.

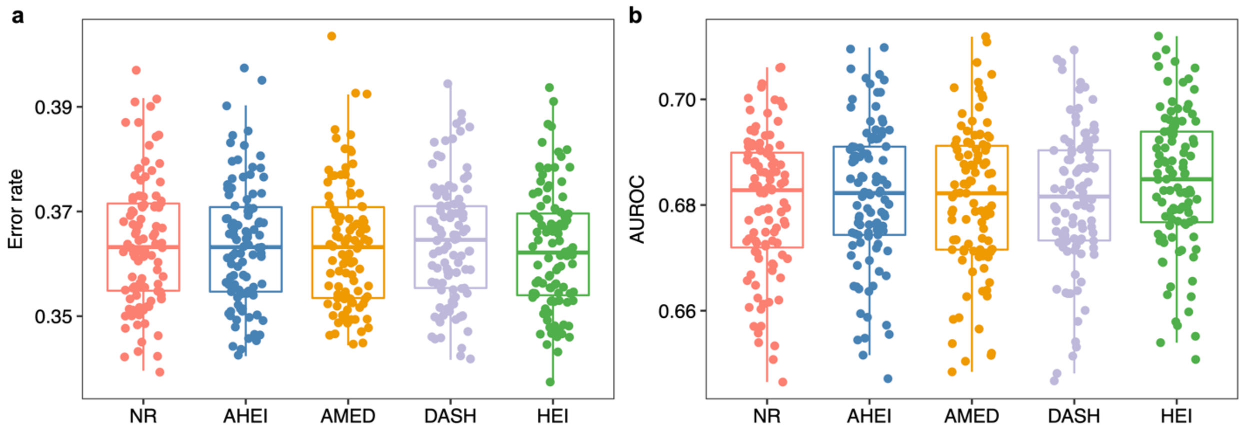

Figure S10: Nutrient redundancy serves as a potential metric to predict healthy aging in HPFS. **a:** Error rate of random forest classifier in the prediction of healthy aging status. **b:** AUROC of random forest classifier in prediction of healthy aging status. The participants are randomly spitted into 80% as the training set and the remaining 20% as the test set. The boxplot represents the performances of 100 independent splits. Boxes indicate the interquartile range between the first and third quartiles with the central mark inside each box indicating the median. Whiskers extend to the lowest and highest values within 1.5 times the interquartile range.

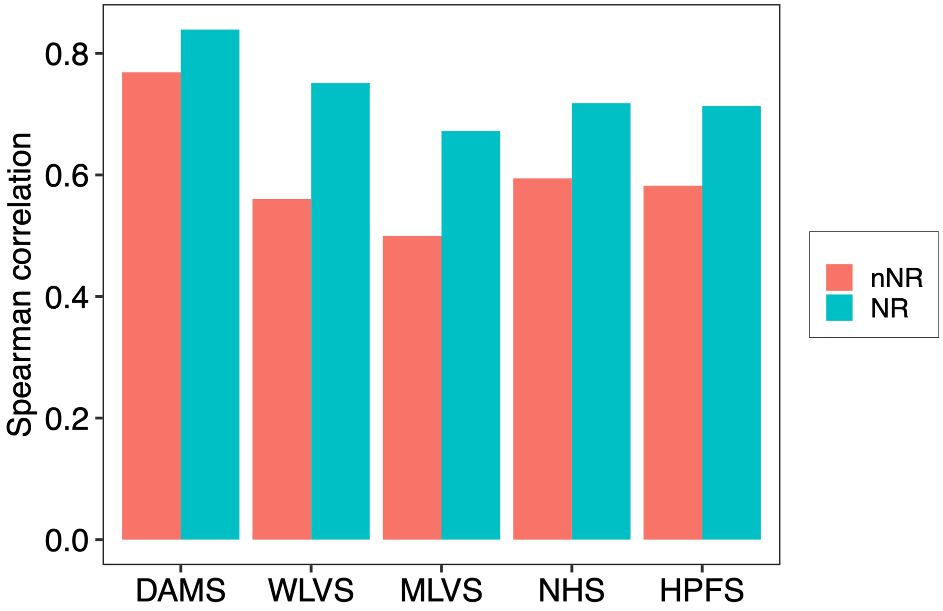

Figure S11: Spearman correlation between the food diversity (FD) and nutritional redundancy (NR) (or the normalized nutritional redundancy: nNR). DMAS (dietary intake data collected using ASA24 dietary assessment tool daily over 17 consecutive days); WLVS (with four ASA24 records within one year); MLVS (with four ASA24 records within one year); NHS (with FFQ administrated every four years and with total eight time points); HPFS (with FFQ administrated every four years and with total seven time points).

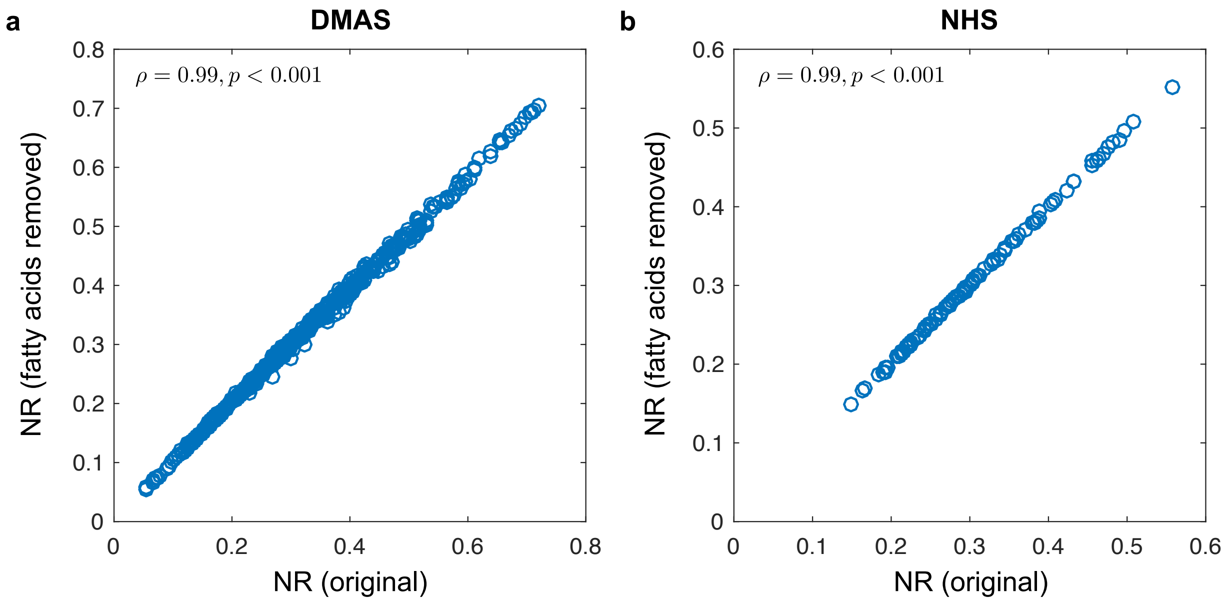

Figure S12: Removing fatty acids does not significantly alter NR. For participants in DMAS (a) and NHS (b, using 100 randomly selected subjects for simplicity), we calculated the Pearson correlation between their NR values calculated from the original food nutrient network (FNN) and the FNN with fatty acids-related nutrients (total fatty acids of saturated, total fatty acids of monounsaturated, and total fatty acids of polyunsaturated) removed. P-values were computed using two-sided Student's t-test.

Characteristics	Healthy agers ($n = 3,491$)	Usual agers ($n = 17,808$)
**Mean age, years**	58.1 (4.9)	62.8 (6.5)
**Educational level (1992), % (n)**		
Registered nurse	57 (1,999)	66 (11,841)
Bachelor's degree	25 (877)	21 (3,776)
Master or doctorate	18 (565)	13 (1,921)
**Marital status (1996), % (n)**		
Windowed	5 (182)	10 (1,792)
Married	86 (3,018)	82 (14,601)
Separated/divorced	7 (267)	8 (1,315)
**Median neighborhood income, $**		
<45302	16 (564)	21 (3,659)
45,302-55,373	17 (606)	20 (3,641)
55,373-66,363	20 (692)	20 (3,553)
66,364-82582	21 (746)	20 (3,506)
>82582	26 (873)	19 (3,414)
**Body mass index, % (n)**		
**<22**	26 (919)	16 (2,886)
**22-24.9**	35 (1,220)	27 (4,856)
**25-29.9**	30 (1,062)	35 (6,212)
**≥ 30**	9 (271)	22 (3,668)
**Multivitamin use, % (n)**	60 (2,108)	61 (10.921)
**Aspirin use, tables per week, % (n)**		
**<1**	67 (2,323)	60 (10,740)
**1-2**	8 (260)	7 (1,217)
**>2**	25 (869)	33 (5,656)
**Pack-years of smoking**		
0	54 (1,881)	48 (8,504)
0-4	13 (464)	11 (1,968)
5-20	20 (701)	20 (3,590)
21-112	12 (406)	20 (3,522)
**Mean physical activity**		
<3.6	11 (367)	21 (3,651)
3.7-9	16 (565)	21 (3,660)
9.1-16.7	21 (721)	21 (3,668)
16.8-30.4	24 (848)	20 (3,578)
>30.5	28 (986)	17 (3,214)
**Energy intake**		
<1,296	17 (601)	29 (3,318)
1,296-1,572	21 (737)	21 (3,683)
1,573-1,830	21 (716)	21 (3,754)
1,831-2,169	22 (748)	21 (3,724)
>2,170	19 (689)	18 (3,329)
**Nutritional redundancy**	0.331 (0.0791)	0.325 (0.0798)
**AMED**	4.814 (1.89)	4.48 (1.86)
**AHEI**	55.5 (11)	53.34 (10.77)
**DASH**	24.58 (4.49)	23.86 (4.48)
**HEI**	74.9 (9.16)	73.08 (10)

Table S1. Characteristics of a subset of NHS participants in 1998 for whom we have their aging status in 2012 from a previous study. Measures in this table were calculated among non-missing values (≤ 5 of data were missing). Data expressed as mean (SD) or percentage.

Table S2. Hazard ratios (95% confidence intervals) of type 2 diabetes according to quintiles of normalized nutritional redundancy (NR) in the Nurses' Health Study (1984-2014) and Health Professionals Follow-Up Study (1986-2016).

	Q1	Q2	Q3	Q4	Q5	P for trend[1]
			NHS			
**Cases/Person-year**	882/187,428	802/187,556	760/187,504	698/187,423	672/187,397	
**Age-adjusted model**	1 (reference)	0.90(0.82,0.99)	0.85(0.77,0.94)	0.75(0.68,0.83)	0.23(0.14,0.38)	<0.001
**Multivariable-adjusted model**[2]	1 (reference)	0.91(0.83,1.01)	0.92(0.83,1.02)	0.89(0.80,0.99)	0.64(0.39,1.06)	0.0827
			HPFS			
**Cases/Person-year**	419/88,643	351/88,705	317/88,792	274/88,848	327/88,844	
**Age-adjusted model**	1 (reference)	0.83(0.72,0.96)	0.75(0.65,0.87)	0.77(0.66,0.89)	0.26(0.14,0.50)	<0.001
**Multivariable-adjusted model**[2]	1 (reference)	0.88(0.76,1.02)	0.82(0.70,0.95)	0.88(0.76,1.03)	0.50(0.260.97)	0.0415

[1]P for trend was calculated using the median value of each quintile (two-sided Chi-square test).

[2]Multivariable-adjusted model adjusted for age (months), total energy intake, ethnicity (white, African American, Asian, others), body mass index (<21.0, 21.0-22.9, 23.0-24.9, 25.0-26.9, 27.0-29.9, 30.0-32.9, 33.0-34.9, or ≥35.0 kg/m^2), smoking status (never smoked, past smoker, currently smoke 1-14 cigarettes per day, 15-24 cigarettes per day, or ≥25 cigarettes per day), alcohol intake (0, 0.1-4.9, 5.0-9.9, 10.0-14.9, 15.0-29.9, and ≥30.0 g/d), hypertension (yes, no), hypercholesterinemia (yes, no), multivitamin use (yes, no), physical activity (quintiles), alternative healthy eating index, total energy (quintiles), family history of myocardial infarction, postmenopausal hormone use (never, former, or current hormone use, or missing), and oral contraceptive use.

Table S3. Hazard ratios (95% confidence intervals) of cardiovascular disease according to quintiles of normalized nutritional redundancy (NR) in the Nurses' Health Study (1984-2014) and Health Professionals Follow-Up Study (1986-2016).

	Q1	Q2	Q3	Q4	Q5	P for trend[1]
			NHS			
**Cases/Person-year**	927/195,361	814/195,530	809/195,422	731/195,286	770/195,193	
**Age-adjusted model**	1 (reference)	0.90(0.82,0.99)	0.90(0.82,0.99)	0.84(0.76,0.92)	0.40(0.25,0.63)	<0.001
**Multivariable-adjusted model[2]**	1 (reference)	0.91(0.83,1.00)	0.93(0.84,1.02)	0.88(0.80,0.98)	0.53(0.33,0.86)	0.0096
			HPFS			
**Cases/Person-year**	848/87,283	774/87,340	735/87,409	765/87,432	811/87,411	
**Age-adjusted model**	1 (reference)	0.91(0.83,1.01)	0.86(0.78,0.95)	0.88(0.80,0.97)	0.58(0.38,0.88)	0.0106
**Multivariable-adjusted model[2]**	1 (reference)	0.93(0.84,1.03)	0.89(0.81,0.99)	0.91(0.83,1.01)	0.69(0.45,1.05)	0.0858

[1]P for trend was calculated using the median value of each quintile (two-sided Chi-square test).

[2]Multivariable-adjusted model adjusted for age (months), total energy intake, ethnicity (white, African American, Asian, others), body mass index (<21.0, 21.0-22.9, 23.0-24.9, 25.0-26.9, 27.0-29.9, 30.0-32.9, 33.0-34.9, or ≥35.0 kg/m^2), smoking status (never smoked, past smoker, currently smoke 1-14 cigarettes per day, 15-24 cigarettes per day, or ≥25 cigarettes per day), alcohol intake (0, 0.1-4.9, 5.0-9.9, 10.0-14.9, 15.0-29.9, and ≥30.0 g/d), hypertension (yes, no), hypercholesterinemia (yes, no), multivitamin use (yes, no), physical activity (quintiles), alternative healthy eating index, total energy (quintiles), family history of myocardial infarction, postmenopausal hormone use (never, former, or current hormone use, or missing), and oral contraceptive use.

Table S4. Hazard ratios (95% confidence intervals) of type 2 diabetes according to terciles of FD in the Nurses' Health Study (NHS, 1984-2014) and Health Professionals Follow-Up Study (HPFS, 1986-2016).

NHS	T1	T2	T3	P for trend[1]
**Cases/Person-year**	1,300/312,451	1,279/312,504	1,235/312,353	
**Age-adjusted model**	1 (reference)	0.98(0.91,1.06)	0.94(0.87,1.02)	0.1579
**Multivariable-adjusted model[2]**	1 (reference)	1.04(0.96,1.12)	1.01(0.93,1.09)	0.8529
**HPFS**				
**Cases/Person-year**	622/147,862	526/147,989	540/147,980	
**Age-adjusted model**	1 (reference)	0.84(0.75,0.95)	0.88(0.78,0.98)	0.0136
**Multivariable-adjusted model[2]**	1 (reference)	0.91(0.81,1.02)	0.99(0.88,1.12)	0.5966

[1]P for trend was calculated using the median value of each terciles (two-sided Chi-square test).

[2]Multivariable-adjusted model adjusted for age (years), ethnicity (white, African American, Asian, others), body mass index (<21.0, 21.0-22.9, 23.0-24.9, 25.0-26.9, 27.0-29.9, 30.0-32.9, 33.0-34.9, or ≥35.0 kg/m^2), smoking status (never smoked, past smoker, currently smoke 1-14 cigarettes per day, 15-24 cigarettes per day, or ≥25 cigarettes per day), alcohol intake (0, 0.1-4.9, 5.0-9.9, 10.0-14.9, 15.0-29.9, and ≥30.0 g/d), hypertension (yes, no), hypercholesterinemia (yes, no), multivitamin use (yes, no), physical activity (quintiles), alternative healthy eating index, family history of diabetes. In NHS, postmenopausal hormone use (never, former, or current hormone use, or missing) and oral contraceptive use were additionally adjusted.

Table S5. Hazard ratios (95% confidence intervals) of cardiovascular disease according to terciles of FD in the Nurses' Health Study (NHS, 1984-2014) and Health Professionals Follow-Up Study (HPFS, 1986-2016).

**NHS**	T1	T2	T3	P for trend[1]
**Cases/Person-year**	1,438/325,560	1,339/325,704	1,274/325,527	
**Age-adjusted model**	1 (reference)	0.95(0.88,1.02)	0.92(0.85,0.99)	0.0307
**Multivariable-adjusted model[2]**	1 (reference)	0.98(0.91,1.06)	0.97(0.90,1.05)	0.4760
**HPFS**				
**Cases/Person-year**	1,374/145,508	1,265/145,700	1,294/145,667	
**Age-adjusted model**	1 (reference)	0.92(0.85,0.99)	0.92(0.86,1.00)	0.0315
**Multivariable-adjusted model[2]**	1 (reference)	0.95(0.88,1.02)	0.97(0.89,1.05)	0.3418

[1]P for trend was calculated using the median value of each terciles (two-sided Chi-square test).

[2]Multivariable-adjusted model adjusted for age (years), ethnicity (white, African American, Asian, others), body mass index (<21.0, 21.0-22.9, 23.0-24.9, 25.0-26.9, 27.0-29.9, 30.0-32.9, 33.0-34.9, or ≥35.0 kg/m^2), smoking status (never smoked, past smoker, currently smoke 1-14 cigarettes per day, 15-24 cigarettes per day, or ≥25 cigarettes per day), alcohol intake (0, 0.1-4.9, 5.0-9.9, 10.0-14.9, 15.0-29.9, and ≥30.0 g/d), hypertension (yes, no), hypercholesterinemia (yes, no), multivitamin use (yes, no), physical activity (quintiles), alternative healthy eating index, family history of myocardial infarction. In NHS, postmenopausal hormone use (never, former, or current hormone use, or missing) and oral contraceptive use were additionally adjusted.

References

1. Chiuve, S. E. *et al.* Alternative dietary indices both strongly predict risk of chronic disease. *The Journal of nutrition* **142**, 1009–1018 (2012).
2. Fung, T. T. *et al.* Mediterranean diet and incidence and mortality of coronary heart disease and stroke in women. *Circulation* **119**, 1093 (2009).
3. Fung, T. T. *et al.* Adherence to a DASH-style diet and risk of coronary heart disease and stroke in women. *Archives of internal medicine* **168**, 713–720 (2008).
4. Liaw, A. & Wiener, M. Classification and regression by randomForest. *R news* **2**, 18–22 (2002).
5. Chen, T. & Guestrin, C. XGBoost: A Scalable Tree Boosting System. *Proceedings of the 22nd ACM SIGKDD International Conference on Knowledge Discovery and Data Mining* 785–794 (2016) doi:10.1145/2939672.2939785.
6. Kuhn, M. Building predictive models in R using the caret package. *Journal of statistical software* **28**, 1–26 (2008).
